# Medical and Philosophical Intelligence

**Published:** 1811-05

**Authors:** 


### MEDICAL AND PHILOSOPHICAL INTELLIGENCE.

Royal Society.—March 1st and 7th.—A paper by Mr. Knight was read, detailing experiments on the manner in which plants shoot forth their radicles. In a former paper Mr K. had proved that gravitation was the chief cause of the descent of the roots of plants; in the present he meant to illustrate by his experiments, how certain plants extend their roots towards water, and others in a direction from water, without sensation or animal sensibility, as erroneously supposed posed by some vegetable physiologists. His experiments established this position sufficiently on mechanical principles, in consequence of the natural inclination to, or aversion from, humidity, according to the particular nature of the plant. He also proved that carrots and parsnips sown in a poor gravelly soil, under which was placed a rich loam, passed through the former, and extended their radicles into the rich loam eighteen inches below the surface.

March 14.—A long and learned paper, by Mr. Baily, was read on the eclipse of the sun predicted by Thales, as recorded by Herodotus. The author entered minutely into the historical and chronological data which support his opinion, and concluded that the eclipse alluded to was an annular one, which occurred in the year 610 before Christ.

---

Wernerian Natural History Society.—At the meeting of the Wernerian Natural History Society at Edinburgh, on the 12th of January last, Professor Jameson read the first part of a series of observations on the geognostic relations of the rocks of the island of Arran. In this memoir he described particularly the granite, gneiss, mica-slate, and clay-slate formations, and also the red sandstone, and porphyry-slate, which occur so abundantly in that island. When describing the *granite*, he stated as a conjecture, that quartz might prove to be an older formation than granite, because the oldest granite contains much quartz, but little mica; and less feldspar than the newer varieties. He pointed out several observations to be made with the view of verifying or refuting this conjecture. In his description of *gneiss* he alluded to the veins of granitic gneiss which traverse it, and which, when the gneiss and granite are in contact, have been represented as veins of granite shooting from the subjacent into the superincumbent rocks. The *red sandstone* the professor appeared inclined to refer to the first or old red sandstone of Werner. When describing its stratification and structure he pointed out the appearances that ought to be attended to, in endeavouring to ascertain the dip and direction of the strata, and cautioned observers against confounding the structure of individual beds with the direction and dip of the strata. The numerous fissures that traverse the sandstone of Arran, and which exhibit every variety of magnitude, direction and dip, afforded apt illustrations of Werner's theory of veins. The *porphyry-slate* the professor described as appearing in the form of overlying conical or irregular tabular-shaped masses, resting on the red sandstone; also in veins traversing granite, sandstone, greenstone, and other rocks. He gave a description of some tabular masses of this rock, accompanied by pitchstone and claystone, contained between strata of sandstone, and which might be confounded with beds, but which he was inclined to consider merely as lateral branches of veins, or as slightly inclined veins.

At the meeting, on the 2d of February, Professor Jameson read the continuation of his mineralogical observations on Arran. He first detailed the geognostic relations of the *flœtz greenstone* of that island. From this account it appeared to occur in overlying masses resting on sandstone, in beds, in sandstone, and in veins that traverse sandstone and other rocks. He then described the various kinds of *pitchstone* that occur in Arran, and seemed to think that one of the varieties might constitute a distinct subspecies of the pitchstone species. The account of its geognostic relations afforded a detail of many curious geognostic appearances; in particular the structure of its veins, and the nature of the interposed tabular mases, having many of the characters of beds, yet appearing to be either nearly horizontal veins, or lateral branches of common veins. The *clay-stone* of Arran, which was next described, appeared to occur in overlying masses, along with porphyry-slate, and also in veins along with pitchstone and porphyry-slate. It would seem that *wacke* and *basalt* are not very frequent or abundant rocks in Arran; but when they are observed, they present the usual appearances and geognostic relations.

From the observations in these two memoirs it appears, that this island contains no *transition* rocks; but is principally composed of *primitive* and *flœtz* rocks. The *alluvial* rocks that occur in the valleys present the usual characters of the rocks of this class.

---

It affords us considerable pleasure to be able to announce the progressive improvement of Medical Science in France. The numerous Societies instituted in that extensive empire, seem to be emulous of the high reputation which has long distinguished many of the professors of medicine, surgery, and chemistry, in this country: the armies abroad, the great hospitals at home, the facility granted to the dissection of dead bodies, and the inspection of patients who die, in whatever rank of life, contribute to increase practical knowledge, in which the French practitioners were formerly extremely deficient. From a recent arrival of various Journals, we find that the spirit of emulation is strongly excited by the proposal of prizes, some of which we subjoin, and as some of our learned readers may be inclined to contend for them, we insert the words of the questions in the original language.

The class of mathematical and physical sciences of the Institute of France offers a gold medal, value 3000 francs, for the following subject—" Donner la theorie mathematique des lois de la propagation de la chaleur, et comparer les resultats de cette theorie avec l'experience." The memoirs must be sent free of expence by the 1st of October, 1811, addressed to the Secretary of the Institute, each bearing a motto or device, which must be repeated with the name of the author in a sealed note attached to the memoir. The result will be published the first Monday in January 1812.

---

*La Société de Médecine de Lyon* has proposed the following question for the subject of the prize (value 300 francs) to be decreed at the public sitting, 1812. " Quelle est l'influence que les maladies organiques des viscères du bas-ventre peuvent exercer sur les viscères de la tête et de la poitrine, soit dans l'exercice de leurs fonctions, soit dans la production de leurs maladies; et quels dangers peuvent résulter, dans la la pratique de la médecine, de l'ignorance et de l'oubli de cette influence?"

The candidates must observe the usual academic forms respecting concealing their names, &c.

The memoirs to be written in French or Latin, and addressed, (free of expence) before the 1st. of January, 1812, to M. Franchet, Secrétaire général de la Société de Medécine de Lyon, place neuve des Carmes, No. 29.

---

*La Société de Médécine-pratique de Montpellier*, has proposed the following prize questions to be adjudged at their public meeting in May 1811.

1. " Quelles sont les maladies chroniques qui passent pour dépendre particulièrement de l'état du cerveau? Peut-on tirer, des ouvertures des cadavres de ceux qui ont succombé à quelques-unes de ces maladies, des inductions propres à en constater l'étiologie; et, dans tous les cas, quelles sont les règles générales ou particulières de traitement dont ces maladies peuvent être susceptibles?"

2. Quelles sont les maladies chroniques, dont les grands systêmes (le vasculaire-sanguin, le vasculaire et le glanduleux-lymphatique, le cellulaire et exhalant, le nerveux et l'osseux) de l'économie animale, sont le siège? Quel est le degré de similitude ou de dissemblance que leurs phénomènes peuvent faire établir entre eux; et quelles sont les règles générales de traitement qui doivent leur être appliquées d'après l'observation?"

The prizes will consist each of a gold medal, value 300 francs.

---

*La Société d'Agriculture de Paris* has proposed the following Prize subjects to be determined at the public sitting, July 15, 1811.

1. Pour la *Culture du Pommier et du Poirier à Cidre*, dans les cantons ou elle n'est pas introduite: 1 prix, 1500 fr. 2 prix, 1000 fr.
2. *Abolition des Jacheres* Medailles d'or.
3. *Introduction d'Engrais dont l'usage est inconnu:* Médailles d'or.
4. *Observations pratiques de Medécine-Veterinaire.* Idem.
5. *Traductions d'ouvrages sur l'agriculture*, qui offrent des observations neuves et des pratiques utiles: Idem.

Prize subjects for 1812.

*Moyens de prévénir la Cécité dans les Chevaux:* prix 400 fr.

*Fabrication de Fromages étrangers*: 1 prix 2000 fr. 2 prix 1000 fr.

*Extraction d'une substance colorante bleue des végétaux cultivés en France*: 1 prix 2000 fr.; 2 prix 1000 fr.

*Multiplication des Abeilles:* 1 prix 800 fr. 2 prix 400 fr.

Prize subject for 1813.

*Machines hydrauliques*, appropriées aux usages de l'agriculture: 1 prix 3000 fr.; 2 prix 2000 fr.; 3 prix 1000 fr.

---

*La Société d'Agriculture du Départment de la Seine*, impressed with the great importance of veterinary science, has solicited communications from veterinarians, on practical subjects connected with their profession. It invites them to perfect the knowledge of the diseases of animals, by carefully dissecting the bodies of those which die die. To promote these desirable views, the Society proposes to distribute annually gold medals, or a sum of money to the amount of 1,200 francs, to those veterinarians who shall furnish the best memoirs, which will be received till the 1st of February each year; they must be signed with the authors' names. The Society also proposed the following subject for a prize of a gold medal, or money, value 1000 francs, to be adjudged at the public meeting in 1811. "To determine by a course of observations, the most frequent causes of blindness in horses, and to indicate the best means of remedying it."

---

*La Société Academique de Médécine*, proposes the following question for the subject of a prize, to be decreed at the ordinary meeting, the second Tuesday in September, 1811. "Quels sont les signes qui indiquent ou contr'indiquent la saigné, soit dans les fièvres intermittentes, soit dans les fièvres continues, designées sous les noms de putrides ou adynamiques, malignes ou ataxiques?" The author who determines this question in a satisfactory manner, will receive a gold medal valued at 300 francs. The memoirs to be written in French or in Latin, and forwarded free of expense, by the 1st of July, to M. Léveillé, Secretary to the Society, rue Neuve-des-Petits-Champs, No. 52.

---

*La Société de Médécine de Besançon*, considering that information is yet wanting on the anatomy, physiology, and pathology of the skin, has offered for the subject of a prize essay, to be adjudged at its public meeting in September, 1811, "l'histoire anatomique, physiologique, sympathique, et pathologique de la peau."

---

*Plumbi acetas* has been given with success in epilepsy, in doses of four and five grains in the day.

The muriate of manganese and the phosphate of manganese produce the same effect as the oxide of iron, and may be given in the same doses.

The phosphate of copper, and the phosphate of silver have been used as purgatives; which effect they produce in doses of a single grain; they have been found excellent hydragogues.

Mercury, given so as to induce ptyalism, continues to be practiced with great success in phthisis in various parts of America.—

*Medical Museum, Philadelphia.*

M. Vitalis, Secretary of the Academy of Sciences, Literature, and Arts, of Rouen, and Professor of Chemistry in the same city, having, in the course of his operations, formed a combination of phosphoric acid and potass, both very pure, and having obtained by suitable evaporation of the liquor, a salt perfectly crystallized, thought that the chemists, who have all announced the incrystallizability (incristallisabilité) of the phosphate of potass, were deceived.

Not wishing to trust to his own experiment alone in controverting what had been asserted on the subject, by the most able chemists, mists, M. Vitalis sent a small quantity of the salt to M. Vauquelin, that he might submit it to experiments. The result of his researches is as follows:

1. The salt is very white, crystallized in prisms, with four equal sides terminated by pyramids with four faces corresponding to the sides of the prism.

2. It is very acid to the taste, and strongly reddens the colour of turnsol: it is not alterable by the air.

3. It precipitates abundantly lime-water in white flakes.

4. Caustic potass does not disengage ammoniac.

5. It precipitates copiously the solution of muriate of platina.

6. It does not give out phosphorus by heat, but it melts in a clear glass which crystallizes, and becomes opaque upon cooling.

7. Thus melted, it does not dissolve in water so readily as before.

8. A portion of this salt having been saturated by potass, and submitted to a spontaneous evaporation, did not crystallize; but was reduced to a kind of viscid fluid like a solution of gum.

From these experiments it clearly follows, that the salt in question is an acid phosphate of potass (phosphate acide de potasse); that thus the opinions of chemists upon the properties of common phosphate of potass, are not affected by the peculiar qualities of this salt; finally, that M. Vitalis has enriched chemistry with a new species of salt which ought to hold a place in the already numerous class of those bodies.—*Annales de Chimie.*

---

Meteoric Stone in India.—The following extract of a letter from Futty Ghur, in the East Indies, dated 21st July last, presents us with an account of a curious phænomenon which has become frequent in Europe and America of late years: "A large ball of fire fell from the clouds, it burnt five villages, destroyed the crops, and some men and women. The ball is still to be seen; it is as hard as a stone. This happened near Shahabad, across the Ganges about thirty miles northward from this place. I have heard nothing further about this, but a vague report."

---

### *On the decomposition of fulminating powders of mercury and silver by charcoal.*

(Journal Général de Médécine, &c.)

Experiments upon living animals had caused the fulminating powders of mercury and silver to be ranked with the most active poisons. M. Pagot Laforet, to whom we owe the knowledge of two of these powders, the fulminating powder of silver and mercury detonating white, and the fulminating powder of silver and mercury detonating grey, has ascertained that very small doses are sufficient to kill in less than half an hour, cats, dogs, hares, and other animals which he had submitted to his experiments. Continuing his remarks concerning these powders, he has found that if we mix pulverized and wet charcoal with the powders that the charcoal attracts tracts their oxygen, and decomposes them, so that they cease to detonate, and they lose their corrosive action, and only yield a metallic taste to the tongue.

M. P. Laforet has endeavoured to make an application of this discovery to the treatment of cases of poisoning by them; he gave to a cat a sufficient dose to occasion death, and as soon as the symptoms of their having taken effect appeared, he made it swallow a great quantity of the powder of charcoal mixed with water in the consistence of thick milk, so as to effect the decomposition of the powders in the stomach. His expectations were not frustrated; he remarked that the symptoms of poisoning ceased immediately, and that the cat suffered no injury from the fulminating powder. He intends to continue his experiments.

### *Vaccination in Glasgow.*

The following statement contains perhaps one of the most decisive proofs of the utility of vaccination which has been submitted to the public. The first column contains the year. The second the number who have died of the small-pox in the city. The third the whole number of deaths in the city. The fourth the number of deaths in the city and suburbs.

| First. | Second. | Third | Fourth. |
|---|---|---|---|
| 1792 | 403 | 1508 | 1912 |
| 1793 | 134 | 1356 | 2190 |
| 1794 | 278 | 1365 | 2445 |
| 1795 | 132 | 987 | 1700 |
| 1796 | 265 | 1327 | 2297 |
| 1797 | 184 | 961 | 1813 |
| 1798 | 231 | 1125 | 2064 |
| 1799 | 179 | 1025 | 2181 |
| 1800 | 224 | 1279 | 2499 |
| 1801 | 159 | 985 | 2096 |
| 1802 | 107 | 825 | 1928 |
| 1803 | 91 | 1158 | 2438 |
| 1804 | 123 | 1011 | 2224 |
| 1805 | 21 | 968 | 2389 |
| 1806 | 15 | 939 | 2280 |
| 1807 | 48 | 1002 | 2463 |
| 1808 | 14 | 1446 | 3265 |
| 1809 | 54 | —— | 2368 |
| 1810 | 23 | 1121 | 2367 |

There are perhaps few towns of the same magnitude where the beneficial effects of vaccination have been more distinctly experienced. None of those jarring opinions, which have disgraced other parts of the kingdom, are known in Glasgow. The profession universally recommend the practice, and the people almost as universally receive it. The few deaths by small-pox, which have occurred, within these last six years, have been exclusively among recent incomers, and the poorest and most wretched of the Highlanders and Irish. It appears from the records of the Vaccine Institution, that, previous to January 1811, upwards of *fourteen thousand five hundred* have been inoculated *gratis.*—*Edin. Jour.*

### *Letter from the National Vaccine Board to the Governors of the Finsbury Dispensary, London, on the bad effects of the practice of inoculating for the Small-Pox, at that Charity.*

*Leicester Square, Oct.* 25, 1810.

SIR,—As the Finsbury Dispensary was instituted from the purest motives of charity and benevolence, it is conceived, that you and the other governors will approve of a communication, intimating, that the practice of the dispensary in one point is producing effects the reverse of your intention.

It is scarcely requisite to inform you that parliament, after fully examining into the merits of the discovery of vaccination, were convinced of the important benefits that would result from its general adoption; and parliament, in consequence, have authorised Government to form a National Vaccine Establishment, in order to extend vaccination to all parts of the empire.

This establishment is placed under the controul of the president and censors, and the master and governors of the royal colleges of physicians and surgeons of London; who, from experience, and authentic information, are fully persuaded of the wisdom of the practice of vaccination, and of the futility of those objections which have been urged against it. They observe, however, with regret, that although vaccination has spread not only through the British empire, but also among foreign nations, with the most beneficial consequences, yet from prejudices artfully kept up, small-pox inoculation is still prevalent among the poor of this metropolis.

About two years ago, the governors of the small-pox hospital took this business into consideration, and, from a conviction of the great mortality which was caused by propagating a contagious and fatal disease, they prohibited their medical officers from inoculating the small.pox, or distributing small-pox matter.

A decrease of deaths from the small-pox was the immediate consequence: but unhappily this fatal malady again rages through the town, and destroys, within the bills of mortality alone, about thirty persons every week.

Upon investigating the sources of this direful pestilence, I have learnt, that besides the pernicious activity of certain private practitioners, the inoculation of small-pox is extensively carried on at the Finsbury dispensary; above a thousand persons having been inoculated there last year, and matter having been distributed for the inoculation of others to an unknown extent.

You are earnestly requested to reflect upon the consequences of thus diffusing among the crowded lanes, courts, and alleys of this populous city, a baneful infection which proves fatal to multitudes, and strikes many with total blindness.

These indisputable facts made an irresistible impression upon the minds of the governors of the small-pox hospital; and as they knew that the inoculation of the small-pox was disapproved of by the most eminent of the medical profession, they were little affected by the misrepresentations of the prejudiced, or by the sophistry of a few who are interested in the propagation of small-pox.

It is to be presumed that when the governors of the Finsbury dispensary deliberate upon this subject, they will decide with equal prudence and humanity. For they are at present, though no doubt unintentionally, opposing a most benevolent measure of parliament; and sanctioning a practice most fatally injurious to the community.

I have the honour to be, &c.

JAMES MOORE, Director.

---

*Report of the Surgeons of the Vaccine Institution of Edinburgh*, 1810.

In reporting to the managers of the Vaccine Institution the state of vaccination for 1810, the surgeons have the satisfaction to mention, that nothing has occurred which can in any degree diminish their belief of the perfect efficacy of the cow-pox, as a preventive of the small-pox.

Since last report, 583 have been vaccinated, making in all 11,108, from the commencement of the practice at the institution.

The Surgeons have, since last report, inoculated with the small pox a great many children, who have been vaccinated eight, nine, and ten years ago—all of whom have been found to resist the infection.

In no instance have they seen any bad effects which could be attributed to vaccination; and, upon the whole, the experience of another year serves to confirm them in their former opinion, that the practice of vaccination is deserving of the highest degree of confidence from the public.

Signed by Wm. FARQUHARSON.
JAMES BRYCE.
ALEXr. GILLISPIE.
*Edinburgh, Jan.* 21, 1811. JOHN ABERCROMBIE.

---

*Letter on Vaccination in Ceylon, from Thomas Christie, Esq. Med. Sup. Gen. to the Editor of the Ceylon Government Gazette.*

Sir,—I beg leave to subjoin, for more general information, an abstract of the number of patients vaccinated in the different districts in Ceylon, during 1809, amounting to 25,697, which added to 103,035, the number vaccinated in former years, makes a total of 128,732 persons who have been officially reported to me, by the respective superintendants and vaccinators, as having regularly passed through the vaccine disease, since its first introduction into this island in 1802, besides a few others inoculated by individuals, not belonging to the vaccination establishment.

Agreeable to the best information I have been able to obtain, the small-pox has not existed in any part of this island, since February 1808, till October last, when the disease was brought to Jaffnapatnam by a country boat from Quilon on the Malabar coast. The contagion contagion spread to a few individuals who had not been vaccinated in the Pettah of Jaffnapatnam, and, by means of a civil prisoner, was introduced into the jail at that place, but its progress there was immediately arrested, by the removal of the infected persons, and the indiscriminate vaccination of all the other prisoners.

By a late report from Mr. Stutzer, Superintendant of vaccination at Jaffnapatnam, it appears that there were only six individuals ill of the small-pox in that district, and it has found its way to no other part of the island, except Putlam, where a coolie from Jaffna was taken ill with small-pox in December last, but has since recovered without communicating the disorder to any person.

The vaccine disease has now been so extensively diffused throughout this island, that while the inoculations continue so numerous as at present, we can have no reason to apprehend that the contagion of small-pox will ever spread epidemically in any part of the British possessions in Ceylon; and its occasional appearance here has the good effect of proving the preservative efficacy of the vaccine, and of rousing the natives from their apathy on the subject, as exemplified at Jaffnapatnam, where 1830 people have been inoculated during the two last months, and amongst them several Bramins, men and women, who had hitherto declined submitting to the operation.

I shall only add, that with a view of proving the permanency of the preservative efficacy of cow-pox, and the continuance of the purity of the virus in this island, Mr. Stutzer has at my request, in November and December last, inoculated with small-pox matter, several patients who had passed through the vaccine disease in 1804, and 1809, all of whom have resisted the contagion.—I have the honour to be, &c.

*Colombo, 24th January* 1810.

---

Theatre of Anatomy.—Lectures on Anatomy, Physiology, Pathology, and Surgery, by Mr. John Taunton, Member of the Royal College of Surgeons of London, Surgeon to the City and Finsbury Dispensaries, City Truss Society, &c.

In this Course of Lectures it is proposed to take a comprehensive view of the structure and economy of the living body, and to consider the causes, symptoms, nature, and treatment of surgical diseases, with the mode of performing the different surgical operations; forming a complete course of anatomical and physiological instruction for the medical or surgical student, the artist, the professional or private gentleman.

An ample field for professional edification will be afforded by the opportunity which pupils may have of attending the clinical and other practice of both the City and Finsbury Dispensaries.

The Summer course will commence on Saturday, May 25th, 1811, at eight o'clock in the evening *precisely*, and be continued every Tuesday, Thursday, and Saturday, at the same hour.

Particulars may be had, on applying to Mr. Taunton, Greville Street, Hatton Garden.

Medical Theatre, Guy's Hospital.—Dr. Thornton wil deliver his Introductory Lecture on Medical Botany, on Wednesday, May 1, at five o'clock in the evening.

---

Mr. Stevenson will commence his Course of Lectures on the Eye and Ear, on Monday the 20th May, at seven o'clock in the evening, at his house, Great Russel-street.

---

Dr. Adams's next Course of Lectures on the Institutes and Practice of Medicine, will commence at the latter end of May, or beginning of June.

---

The Editor of the Monthly Magazine has announced his design to publish early in May, all the Communications in a separate pamphlet which have been made to him by various persons relative to the uses of Stramonium in Asthma. The collection will contain other papers besides those published; a description of the plant, and a coloured engraving.

---

Darwin's Zoonomia has recently been translated into French by J. F. Kluiskens, and published in five volumes, 8vo.

---

A French edition of the Medico-Chirurgical Transactions is nearly ready for publication at Paris; where the character of the work ranks very high.

---

The Rev. John Rudd, F. L. S. and President of the new Literary and Philosophical Society of Preston, has in considerable forwardness, a Botanist's Guide through Lancashire, in which all the Plants, indigenous to the County, will be enumerated, and their habitats accurately given.

---

Mr. Benjamin Travers, demonstrator of Anatomy at Guy's Hospital, has in the Press, an Inquiry concerning injuries to the intestinal canal, illustrating the treatment of wounds penetrating into that canal, and of strangulated Hernia.

---

Dr. Nott, of Bristol, is printing a Posological Companion to the London Pharmacopœia.

---

Dr. Underwood has in the Press, a new Edition, making the 6th, with considerable additions, of his Treatise on the Diseases of Children.

---

Dr. Adams is preparing a Syllabus of his Lectures, for the assistance of those hearers to whom the doctrines of Mr. Hunter are less known, or who are unaccustomed to apply them to medicine.

---

Professor Boyer is preparing an extensive work on Surgery.

M. Deschamp is preparing for the press a work on Aneurism.

M. Pelletan

M. Pelletan, surgeon of the Hospice d'Humanité at Paris, has in the Press two volumes of Clinical Cases and Observations, illustrated by numerous Engravings.

